# HIV prevalence among female sex workers, drug users and men who have sex with men in Brazil: A Systematic Review and Meta-analysis

**DOI:** 10.1186/1471-2458-10-317

**Published:** 2010-06-07

**Authors:** Monica Malta, Monica MF Magnanini, Maeve B Mello, Ana Roberta P Pascom, Yohana Linhares, Francisco I Bastos

**Affiliations:** 1 Social Science Department, Sergio Arouca School of Public Health, Oswaldo Cruz Foundation, Rio de Janeiro, Brazil; 2 Institute of Public Health Studies, Federal University of Rio de Janeiro, Rio de Janeiro, Brazil; 3 Health Information Laboratory, Center for Scientific and Technological Information, Oswaldo Cruz Foundation, Rio de Janeiro, Brazil; 4 Surveillance Unit, Brazilian National STD/AIDS Program, Brasilia, Brazil

## Abstract

**Background:**

The Brazilian response towards AIDS epidemic is well known, but the absence of a systematic review of vulnerable populations ─ men who have sex with men (MSM), female sex workers (FSW), and drug users (DU) remains a main gap in the available literature. Our goal was to conduct a systematic review and meta-analysis of studies assessing HIV prevalence among MSM, FSW and DU, calculating a combined pooled prevalence and summarizing factors associated the pooled prevalence for each group.

**Methods:**

Nine electronic databases (MEDLINE via PubMed, EMBASE, Cochrane CENTRAL, AIDSLINE, AMED, CINAHL, TOXNET, SciELO, and ISI-Web of Science) were searched for peer-reviewed papers published in English, French, Spanish or Portuguese, from 1999 to 2009. To be included in the review, studies had to measure HIV prevalence and/or incidence as the primary outcome among at least one specific population under analysis.

**Results:**

The studies targeting the three populations analyzed mostly young participants aged 30 years or less. Among FSW, eight studies were selected (3,625 participants), consistently identifying higher condom use with sexual clients than with occasional and stable partners. The combined HIV prevalence for FSW was 6.2 (95% CI: 4.4-8.3). Ten studies targeting MSM were identified (6,475 participants). Unprotected anal intercourse was commonly reported on those studies, but with great variability according to the nature of the relationship - stable vs. occasional sex partners - and sexual practice - receptive vs. insertive anal sex. Pooled HIV prevalence for MSM was 13.6 (95% CI: 8.2-20.2). Twenty nine studies targeting DU were identified (13,063 participants). Those studies consistently identified injection drug use and syringe/needle sharing as key predictors of HIV-infection, as well as engagement in sex work and male-to-male sex. The combined HIV prevalence across studies targeting DU was 23.1 (95% CI: 16.7-30.2).

**Conclusions:**

FSW, MSM and DU from Brazil have a much risk of acquiring HIV infection compared to the general population, among which HIV prevalence has been relatively low (~0.6%). Those vulnerable populations should be targeted by focused prevention strategies that provide accurate information, counseling and testing, as well as concrete means to foster behavior change (e.g. access to condoms, drug abuse treatment, and clean syringes in the case of active injecting drug users), tailored to gender and culture-specific needs. Programs that provide these services need to be implemented on public health services throughout the country, in order to decrease the vulnerability of those populations to HIV infection.

## Background

HIV/AIDS is the largest pandemic ever faced by humankind, with over 30 million people living with HIV/AIDS (PLWHA) worldwide. Over 95% of new infections since 2003 have been reported in low and middle-income countries. According to the UNAIDS, "for every two people who start taking antiretroviral drugs, another five become newly infected. Unless we take urgent steps to intensify HIV prevention we will fail to sustain the gains of the past few years, and universal access will simply be a noble aspiration."[1].

Among the 135 low- and middle-income countries mentioned in a recently released report, UNAIDS estimates that 97 (71.8%) countries have low-level or concentrated HIV/AIDS epidemics [2]. In a concentrated epidemic, HIV spreads rapidly in one or more specific subpopulations, but its spread has been relatively modest in the general population. In these contexts the networks of at-risk populations have a key role in the epidemic dynamics. The future course of the epidemic is determined by the nature and intensity of the interactions between subpopulations with high infection rates and the general population [3-5]. To reduce the likelihood that a low-level or concentrated epidemic may become a generalized epidemic, prevention programs should focus on potential epidemiological bridges, such as the sex partners of injecting drug users (IDU), female sex workers (FSW) or truck drivers [6,7].

Most countries from Latin America have been affected by concentrated HIV/AIDS epidemics and HIV infection rates in this region have changed little in the past decade. HIV transmission is occurring primarily among men who have sex with men - MSM [8,9], and (to a lesser extent) among FSW, IDU and non-injection drug users - NIDU [10].

In Brazil, AIDS prevalence among the general population has been low in recent years: 0.6% -- 0.4% among women and 0.8% among men [11]. The Brazilian commitment towards building and sustaining a timely and comprehensive HIV/AIDS prevention and treatment program can potentially pave the way for other developing countries with similar concentrated epidemics [12].

Brazil was the first middle-income country to provide free and universal access to highly active antiretroviral therapy (HAART), laboratory monitoring and clinical care at no cost at the point of health care delivery to any eligible patient, since 1996 [13,14]. As of the end of 2009, approximately 200,000 patients were receiving HAART in Brazil, all of them monitored by regular CD4+ count and viral load tests (and genotyping, if it is necessary), making it the most comprehensive HIV treatment initiative implemented by a middle-income country, worldwide [15-17]. Brazil has also implemented prevention initiatives targeting both the general population and different at-risk populations [18].

Despite being one of the first countries to implement free and universal access to HAART, together with a concerted array of prevention strategies, only recently Brazil has implemented comprehensive biological and behavioral surveillance surveys (BSS) to monitor trends of HIV infection rates (together with other sexually transmitted infections and blood-borne infections) among the most at-risk groups: MSM, FSW, IDU and NIDU.

In the absence of reliable biological and BSS data on most at-risk populations, HIV/AIDS prevalence rates represent the best information about the current status of the HIV epidemic in the country [1]. This information is vital to inform health planning, resources allocation and might also be an important tool for advocacy and the elaboration of future scenarios [1]. Trying to contribute for a better understanding of the HIV/AIDS epidemic in Brazil, a country with a concentrated epidemic, we conducted a systematic review followed by meta-analysis. Our study summarizes the peer-reviewed literature on HIV prevalence among populations under increased risk of acquiring HIV infection in Brazil and presents a pooled HIV prevalence for the following populations: FSW, MSM and IDU/NIDU.

## Methods

In planning a systematic review and meta-analysis, we reviewed standard guidelines to conduct and report meta-analysis studies, which included the Consolidated Standards of Reporting Trials - CONSORT [19,20], the Quality of Reporting of Meta-analyses - QUOROM [21], the Meta-analysis of Observational Studies in Epidemiology (MOOSE) Group [22], and the Transparent Reporting of Evaluations with Nonrandomized Designs - TREND [23]. As only observational studies rather than clinical trials have been found, the MOOSE recommendations were used to conduct and report the findings from the meta-analysis, while the TREND checklist (Version 1.0) was used as a guide for data abstraction. The same strategy was used by our group in previous reviews [24,25].

### Search Strategy

Search strategies were developed using systematic automated and manual searches. First, we conducted a comprehensive automated search of nine electronic bibliographic databases -- including MEDLINE via PubMed, EMBASE, Cochrane CENTRAL, AIDSLINE, AMED, CINAHL, TOXNET, SciELO, and ISI-Web of Science. Such databases were searched for the period extending from January 1999 to June 2009; except for AIDSLINE, which was searched from 1996 up to 2000, when the inclusion of new citations was discontinued. This search combined standardized search terms (keywords and medical subject heading terms ─ MESH) that reflect key domains: (a) HIV/AIDS, (b) prevalence or incidence, (c) location (Brazil), and (d) target populations (i.e., FSW, MSM, IDU or non-injection drug users). Citations that intersect all four domains were downloaded into the study database.

To reduce publication bias and gaps in the automated search, we implemented four supplementary search strategies to identify additional studies. First, we searched the published conference abstracts from HIV/AIDS and STD conferences using the same domains as the automated search. Second we searched the National Institutes of Health's Computer Retrieval of Information on Scientific Projects (CRISP) database http://projectreporter.nih.gov/reporter.cfm  and the Brazilian online database on researchers and their respective publications, research projects and graduate students http://lattes.cnpq.br to identify researchers working in the field of HIV/AIDS. Third, we contacted authors of selected papers to obtain additional data on upcoming publications. Finally, we reviewed the reference lists of all selected studies for additional citations. All studies identified through these procedures that met our eligible criteria were entered into the study database.

To be included in the review, studies should have measured HIV prevalence and/or incidence as the primary outcome among at least one of the specific populations under analysis.

### Study Selection and Data Extraction

Using a predefined protocol, two investigators (MM, YL) extracted data from peer-reviewed papers measuring/estimating HIV prevalence or incidence among the selected populations and independently assessed their eligibility. Using standardized coding forms, each selected paper was coded for study characteristics (study date, location, study design [cross-sectional, cohort], recruitment setting, participant characteristics (age, gender, race/ethnicity, condom use, needle/syringe sharing), and factors found to be associated with HIV-infection. For those factors multivariable analyses were carried out.

### Statistical Analysis

Standard meta-analytic methods were employed [26,27]. We chose a random-effects model for aggregating individual effect sizes because it provides a more conservative estimate than a fixed-effects model of variance. This approach generates more accurate inferences due to the fact it recognizes the selected studies as a sample of all potential studies and incorporates between-study variability in the overall pooled estimation [28,29]. HIV prevalence and the crude (non-adjusted) proportion of participants recorded as HIV-positive by each study were used to pool the overall proportion, using the DerSimonian-Laird random-effects method [30,31].

The I^2 ^index was calculated as a measure of the overall variation in prevalence that was attributable to between-study heterogeneity [32,33]. Higgins and Thompson [32] proposed a tentative classification of I^2 ^values with the purpose of helping to interpret its magnitude. Thus, percentages of around 25% (I^2 ^≤ 25), 50% (I^2 ^≈ 50), and 75% (I^2 ^≥ 75) were interpreted as low, medium, and high heterogeneity, respectively. According to a recent review [34], the I^2 ^index assesses not only heterogeneity in a meta-analysis but also the extent of that heterogeneity. It is considered a more appropriate procedure than the Q test in assessing whether there is true heterogeneity among the studies in a meta-analysis [33]. Experts have demonstrated that the I^2 ^index exhibits higher power with a larger number of studies (>20) with an average sample size higher than 80 individuals [34].

We anticipated a large between-study heterogeneity (I^2 ^≥ 75), considering the characteristics of subpopulations investigated and the study designs. According to standard meta-analysis guidelines, when observational studies are pooled, heterogeneity of populations (e.g. IDU and NIDU), and of design (e.g. cross-sectional vs. cohort studies) are expected [22,35].

Publication bias was examined through the use of a funnel plot [36], and funnel plot asymmetry was further tested by using Egger's method [37]. Sensitivity analyses were performed to assess whether there were potential heterogeneity sources and studies that may bias the analyses. Studies potentially influencing heterogeneity were therefore removed from the analyses and results compared.

One forest plot was drawn for each population group (FSW, MSM and drug users [including IDU and NIDU]). Forest plots were sorted according to years data were collected (starting with the older studies) to illustrate the HIV-prevalence, its 95% confidence intervals (CI) and the overall DerSimonian-Laird pooled estimate. For studies addressing drug users we also conducted a mixed-effects meta-regression model to assess the underlying reasons for between-study heterogeneity. The small number of eligible studies targeting FSW and MSM, and the absence of key information that could influence between studies heterogeneity precluded meta-regression analysis for those populations [38].

For studies on drug users, results from univariate analyses with p-values ≤ 0.20 were included in the multivariable analysis. The following covariates were included in the meta-regression multivariable model: incarceration (currently incarcerated vs. non-incarcerated participants), type of drug using population (IDU vs. NIDU), region where the study was conducted (Brazilian southern region vs. other regions), year of data collection (until 2001 or latter), and recruitment site (street vs. health centers' recruited participants). According to Hacker et al. [39,40] IDU have played a central role in the HIV/AIDS subepidemic in the southern border of Brazil, therefore we dichotomized studies according to Brazilian region (south vs. other regions). Recently, Bastos et al. [10,18] described that AIDS incidence has been declining in Brazil since 2002. Therefore, we dichotomized studies according to data collection period as well.

Analyses were conducted using Stata version 10.0 (StataCorp, College Station, Tex) and graphics were generated using StatsDirect version 2.5.2 (StatsDirect Ltd, Cheshire, England).

## Results

### Female sex workers

**Figure 1:**
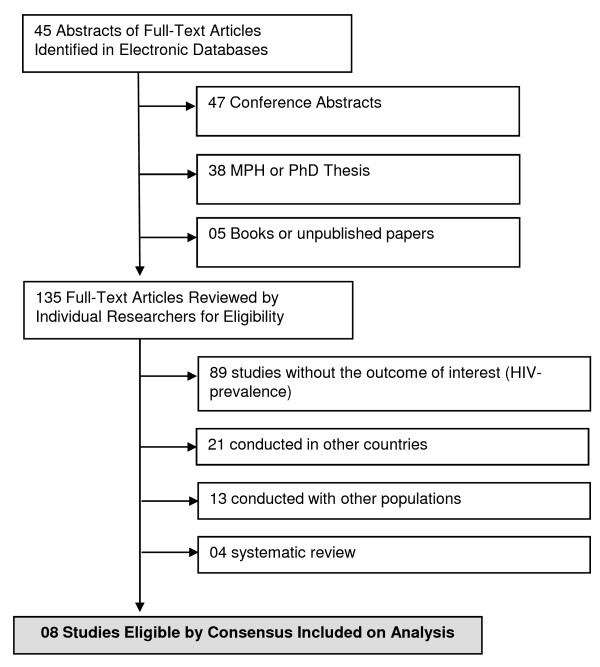
**Flow Diagram of Studies Included in Analysis, CSW**.

In the initial searches, 135 studies were selected (45 peer-reviewed papers and 90 additional studies). Of these, there was perfect agreement between reviewers on the exclusion of 89 behavioral surveys without information on HIV seroprevalence. In a second screening, 21 studies conducted in other countries (rather assessing Brazilian expatriates) were excluded. Agreement between reviewers was also perfect on the second screening. A third screening excluded 13 studies, primarily because authors did not stratify results according to the actual engagement of the interviewees in commercial sex. A final screening excluded four other reviews. Agreement on the two final screenings was also perfect. We thus included eight eligible reports for full data extraction [41-48] (Figure 1).

#### Study characteristics and major study findings

Selected studies analyzed 3,625 FSW (range: 90-2712; median: 143.5 participants), seven of them cross-sectional studies and one of them a retrospective cohort study. The largest study was conducted in 9 Brazilian cities and included 2,712 participants [47]. The majority of participants were young women aged 30 years or less. However, self-reported condom use across selected studies presented a great variability overall and for each specific study. Variability was higher with sexual clients than with occasional and stable partners. According to the largest study conducted with FSW in Brazil, condom use was more than threefold greater when we compared sexual intercourse among sexual clients and stable partners: 67.3% vs. 19.2% (Table 1). Only three studies conducted multivariable analyses, which precluded additional pooled analysis of factors putatively associated with the outcome (HIV-prevalence).

**Table 1:** Characteristics of selected studies and AIDS prevalence among female sex workers from Brazil, 1998-2009.

Source	N	State	Design (Period)	Characteristics of Study Population		Sexual behavior and HIV infection		
				
				Age [mean (range)]	Ethnicity (%)	Condom use (%)	HIV prevalence	Variables associated with prevalent HIV
Barroso et al. [41]	93	Rio de Janeiro	Cross-sectional (2006)	NA	NA	NA	12.9%	NA

Benzaken et al. [42]	114	Amazon	Cross-sectional (2006)	29 (IQR^1 ^: 22-38)	NA	NA	2.6%	NA

Lacerda et al. [43]	175	São Paulo	Cross-sectional (2005-06)	29 (18-62)	Caucasian: 53.1% Black: 8.0% Mulatto: 34.9% Others: 4.0%	Always use condom with client: 73.1%	5.7%	NA

Dutra & Vasques [44]	154	Amazon	Cross-sectional (2005-06)	30.3 ± 8.8	Caucasian: 17,1% Black: 13,3% Mulatto: 69,6%	Always use condom: With stable partner: 36.3% With occasional partner: 25.2%	2.6%	NA

Trevisol & Silva [45]	90	Santa Catarina	Cross-sectional (2003-04)	27 ± 5.2	Caucasian: 85.6% Black: 4.4% Mulatto: 10.0%	Condom use: Always: 16.7% Sometimes: 77.8% Never: 5.6%	6.7%	≤ 2 clients/day (p = 0.008) Infrequent condom use (p = 0.015) Use of inhalants (p = 0.053)

Benzaken et al.[46]	147	Amazon	Cross-sectional (2000)	25.5 (12-54)	NA	NA	0.0%	NA

Brazilian Ministry of Health [47]	2712	9 states	Cross-sectional (2000-01)	Age group: 17-19: 8.1% 20-24: 25.0% 25-29: 20.7% 30-39: 26.8% 40-49: 14.0% ≥50: 5.3%	NA	Always used condom on previous 6 months: With sexual clients: 67.3% With stable partner: 19.2%	Overall: 6.1% Intervention group: 6.6% Control group: 5.6%	Injection drug use: RR: 6.77 (3.44 - 13.17) IDU partner: RR: 2.70 (1.89 - 3.85) Syphilis coinfection: RR: 3.56 (2.00 - 6.29) HCV coinfection: RR: 11.26 (7.28 - 17.40)

Pires & Miranda [48]	140	Espírito Santo	Retrospective Cohort (1993-96)	25.9 ± 6.8	NA	Always: 31.3% Sometimes: 52.0% Never: 16.7%	8.6%	Injection drug use (p = 0,031) Syphilis coinfection (p = 0,014).

^1 ^IQR - Interquartile range

According to Trevisol and Silva [45], covariates independently associated with HIV prevalence included: "having more than two sexual clients per day", "frequent use of inhalants" and "inconsistent condom use". Pires and Miranda [48] identified as key risk factors: "syphilis" and "injection drug use".

The Brazilian Ministry of Health study [47] identified "injection drug use" and "having an IDU partner" as covariates independently associated with the outcome, as well as syphilis and HCV infection. We kept the risk factors as described in the original paper, despite the fact that, for instance, HCV infection should be rather viewed as a biomarker of underlying risk behaviors (e.g. injecting drug use), rather than an independent risk factor for HIV infection (Table 1).

#### Meta-Analysis

After conducting a sensitivity analysis, we decided to present the combined HIV prevalence separately, with the inclusion or exclusion of one "outlier". The identification of this study as an 'outlier' was not based on an a priori statistical criterion (e.g. two standard deviation of the mean), but rather on a thorough evaluation and comparison of the study characteristics with all selected studies. The inclusion of this study significantly decreased the pooled prevalence, and increased the between-study heterogeneity (I^2 ^= 81.9 *vs. *I^2 ^= 56.8). The "outlier" study, conducted by Benzaken and colleagues [46], was developed in a municipality with less than 100,000 inhabitants located in the Tropical rain forest, in the state of Amazon, and did not identify a single HIV-positive participant. This absence of HIV-positive participants is likely due to the study's sampling frame. Additional studies conducted with FSW from the same region identified a prevalence of 2.6% [42].

**Figure 2:**
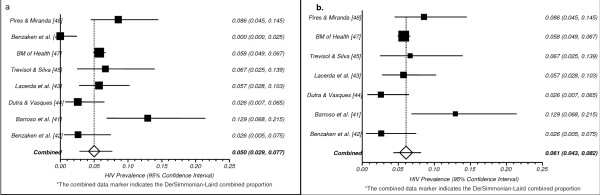
a. Pooled Proportion of HIV Prevalence among CSW, all studies* b. Pooled Proportion of HIV Prevalence among CSW, 7 studies*.

The combined HIV prevalence across all studies was 5.1 (95% CI: 2.9-7.8), while the pooled HIV prevalence, excluding this specific study, was 6.2 (95% CI: 4.4-8.3) (see Figures 2a** and **2b, respectively). The absence of explicit data on key parameters and/or covariates that might be associated with the between-studies heterogeneity (e.g. condom use) precluded meta-regression modeling.

### Men who have sex with men

**Figure 3:**
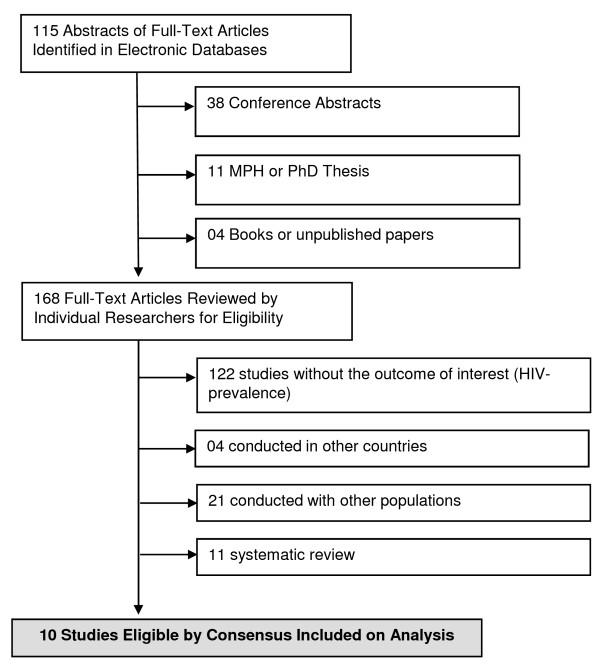
**Flow Diagram of Studies Included in Analysis, MSM**.

In the initial searches, 168 studies were selected (115 peer-reviewed papers and 53 additional studies). Of these, there was perfect agreement between reviewers on the exclusion of 122 behavioral surveys which did not measure HIV seroprevalence. In a second screening, 4 studies conducted in other countries were excluded. Agreement between reviewers was also perfect on the second screening. A third screening excluded 21 studies, primarily because authors did not stratify results according to homosexual practices. A final screening excluded 11 reviews. Agreement on the two final screenings was also perfect. We thus included ten studies for full data extraction [49-58](Figure 3).

#### Study Characteristics and major study findings

Selected studies analyzed 6,475 men who have sex with men (range: 187-1,082; median: 639.5 participants), seven of them cross-sectionals studies and three prospective cohort studies. Szwarcwald et al. [51] conducted a large cross-sectional study with Brazilian military conscripts, while Ferreira et al. [50] analyzed a large sample of MSM who were also IDU. The other studies were conducted in large urban areas from Brazilian South and Southeast regions [49,52-58].

The majority of participants were young MSM aged 30 years or less. Unprotected anal intercourse was commonly reported in the selected studies, but presented a great variability according to the nature of the relationship with their partner(s) - stable vs. occasional sex partners - and sexual practice - receptive vs. insertive anal sex (Table 2).

**Table 2:** Characteristics of selected studies and AIDS prevalence among men who have sex with men from Brazil, 1998-2009.

				Characteristics of Study Population		Sexual behavior and HIV infection		
**Source**	**N**	**State**	**Design (Period)**	**Age**	**Ethnicity (%)**	**Condom use (%)**	**HIV prevalence**	**Variables associated with prevalent HIV**

Tun et al. [49]	658	Campinas	Cross-sectional (2006)	Median: 23	NA	Unprotected sex	Overall sample: 7% (5-11%)	NA
						MSM engaged in sex work:		
						Insertive anal sex: 21.0%	MSM engaged in sex work: 14%	
						Receptive anal sex: 22.0%		
						Vaginal sex: 23.0%		
						MSM not engaged in sex work:	MSM not engaged in sex work: 6%	
						Insertive anal sex: 5.0%		
						Receptive anal sex: 5.0%	Participants between 14-19 years: 4%	
						Vaginal sex: 6.0%		
						Unprotected receptive anal sex with at least one partner: 30% (CI: 26-35%)		
						Unprotected receptive anal sex with ≥ 2 partners: 7% (4-10%)		
						Unprotected anal/vaginal sex with female, previous 2 months: 75% (37-91%)		

Ferreira et al. [50]	187^2^	Brazil	Cross-sectional (2000-01)	28 ± 8.2	Non white: 51.1%	Always use condom: 36.4%	51.9%	NA

Szwarcwald et al.[51]	898^3^	Brazil	Cross-sectional (2002)	17-20 years: 94.0%	NA	Always use condom: 34.1%	0.564 (0.278-0.850)	NA

Schechter et al. [52]	200	Rio de Janeiro	Prospective cohort (1998-2001)	28	White: 47.0%	Unprotected anal sex, previous 6 months:	NA^5^	NA
					Black: 21.0%	PEP^5 ^: 47.1%		
					Mulatto: 7.0%	Non-PEP: 36.4%		
					Others: 26.0%			

Barcellos et al. [53]	461^6^	Porto Alegre	Cross-sectional (1996)	NA	NA	NA	24.1%	NA

Carneiro et al. [54]	621	Belo Horizonte	Cross-sectional (1994-1999)	Mean: 28	NA	NA	9.8%	Unprotected sex w/occasional partner: AOR^7 ^: = 3.7 (: 1.1 -11.9) Receptive anal sex w/occasional partner: AOR = 2.8 (0.9- 8.9) Black vs. Non-black: AOR = 3.4 (1.3-10.6)

Sutmöller et al.[55]	1165	Rio de Janeiro	Prospective cohort^8 ^(1994-1998)	Range: 18-50	NA	NA	24.1%	NA

Carneiro et al. [56]	470	Belo Horizonte	Cross-sectional (1994-1999)	26.9 ± 6.8	Mulatto: 51.8% White: 40.9% Black: 7.3%	Unprotected anal sex with occasional partner: 41.3%^9^	NA	NA

Brazilian Ministry of Health [57]	642	São Paulo	Cross-sectional (1994-1999)	28	NA	Stable partner	8.8%	NA
						Receptive anal sex: 33.9%		
						Insertive anal sex: 36.0%		
						Occasional partner		
						Receptive anal sex: 13.7%		
						Insertive anal sex: 15.3%		

Harrison et al. [58]	849	Rio de Janeiro	Prospective cohort^10 ^(1995-97)	HIV+: 28.8	HIV+: 63.6%	Unprotected receptive anal sex:	11.7%	Penile or anal lesion (p < 0.01)
				HIV-: 28.2	HIV-: 50.7	HIV+: 59.6%		Hepatitis B seropositivity (p < 0.01)
						HIV-: 43.6%		
						Unprotected insertive anal sex:		History of syphilis (p < 0.01)
						HIV+: 34.3%		
						HIV-: 30.8%		

^2 ^All MSM and injection drug users (IDU)

^3 ^MSM sample size among 30,970 military conscripts

^4 ^PEP: *Post-sexual Exposure Prophylaxis*

^5 ^*Only incidence rates are available*

^6 ^*Homo and bisexuals included*

^7 ^AOR: *Adjusted Odds Ratio*

^8 ^Baseline data

^9 ^Among 265 participants reporting anal sex

^10 ^Baseline data

Carneiro et al. [54] identified almost a four-fold increase in HIV prevalence among those men who reported unprotected anal intercourse with occasional partners (adjusted odds ratio - AOR: 3.7) and a greater than three-fold risk of being infected among those of black ethnicity (AOR: 3.4) The authors did not further explore the association between black ethnicity and HIV infection, compromising inferences about the proximal risk factors putatively involved in such association (e.g. attitudes toward safer behaviors, inconsistent use of condoms, etc.).

According to the study conducted by Harrison et al. [58], factors found to be independently associated with seroconversion in their sample of MSM were: "age < 25 years", "sex at the first encounter in the previous six months", and a medical history for the following infections: HBV, gonorrhea or condyloma. (Table 2).

#### Meta-Analysis

Two studies were excluded from our pooled analysis because their outcome was HIV-incidence instead of prevalence [52,56]. After conducting a sensitivity analysis, we decided to exclude two outlier studies from the pooled HIV-prevalence, since both analyzed very specific subgroups of MSM that are very different from the population accessed by all other selected studies. The study by Szwarcwald et al. [51] was conducted with Brazilian military conscripts, a population with distinct characteristics from those studied in the other papers, and found a very low HIV-prevalence (0.564, 95% CI: 0.278-0.850). The primary aim of this large survey was not to assess the behavioral characteristics of young gay men (aged 17-18 years old), but rather to assess the profile of young conscripts as a whole. The small number of self-declared gay or bisexual conscripts precluded further analysis for this specific subpopulation.

**Figure 4:**
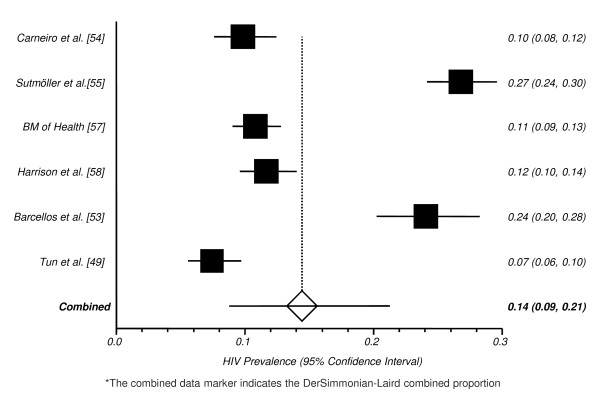
**Pooled Proportion of HIV Prevalence among MSM**.*

On the other hand, the study by Ferreira et al. [50] was conducted among male injection drug users who also reported homosexual behaviors, and found a very high HIV prevalence (51.9%). Even after excluding those studies, a large between-study heterogeneity remained (I^2 ^= 97.1%), and funnel plot asymmetry was evident. In the sensitivity analysis we were not able to attribute the identified asymmetry to any single study. Studies were conducted using different designs (cohort vs. cross-sectional surveys), over many years (from 1994 to 2006) and in very different settings (research centers, public health facilities, and NGOs), therefore compromising the reliability of our pooled analysis. Due to the small number of studies and its great variability, we were unable to conduct subgroup analyses. Those caveats should be also considered in the analysis of pooled HIV prevalence from the remaining 6 studies: 13.6 (95% CI: 8.2-20.2, Figure 4). The absence of key covariates and/or parameters that could explain the between-studies heterogeneity precluded meta-regression modeling.

### Injection and non-injection drug users

**Figure 5:**
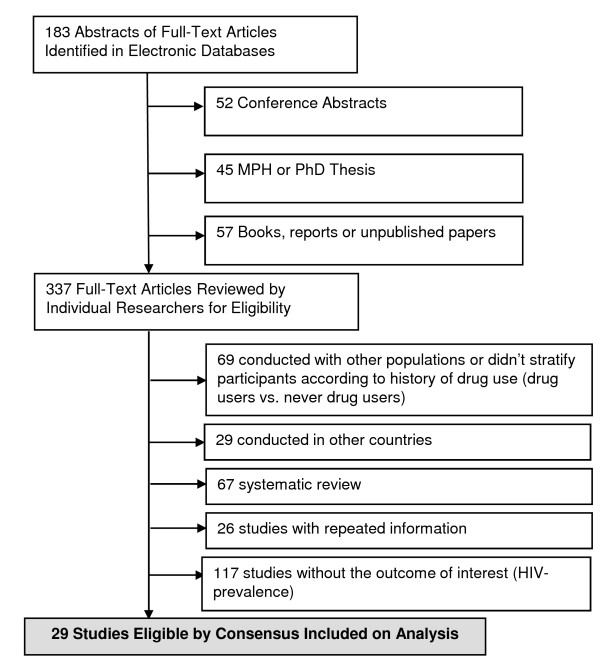
**Flow Diagram of Studies Included in Analysis, IDU and DU**.

In the initial searches, 337 studies were selected (183 peer-reviewed papers and 154 additional studies). Of these, there was perfect agreement between reviewers on the exclusion of 117 behavioral surveys without information on HIV seroprevalence. In a second screening, 29 studies conducted in other countries were excluded. Agreement between reviewers was also perfect on the second screening. A third screening excluded 69 studies, primarily because authors did not stratify results according to the actual use of substances. The remaining 67 reviews were also excluded, as well as 26 studies which provided information already extracted from more recently published studies on the same population, by the same research group (i.e. excluded studies published by the same research group describing partial findings). Agreement on the three final screenings was perfect. We thus included twenty nine reports for full data extraction [39,53,59-85] (Figure 5).

#### Study Characteristics and major study findings

Selected studies analyzed 13,063 injection and non-injection drug users (range: 89-1544; median: 250 participants). The vast majority of them were cross-sectionals studies (n = 25), but there were two prospective cohorts and two case-control studies. Two manuscripts reported findings from a single multicenter study conducted in Brazil with IDU population - AjUDE Brasil I and II [65,67]. The vast majority of selected studies were conducted with street drug users from large urban cities located in Brazilian South, Southeast, and the southernmost part of the Northeast region [39,53,59-64,66,68-85]. Four studies were conducted with incarcerated populations, and their findings were stratified according to previous drug use [60,72,78,83].

Most participants were young NIDU/IDU aged 30 years or less. Needle sharing was commonly reported by the studies under analysis, but under a great variability. According to the study conducted by Albuquerque [80], 15.4% of IDU shared syringe/needles in the previous six months. Guimarães et al. [77] identified that 92.3% of HTLV-infected IDU have shared needle/syringes. Injection drug use has been declining in recent years as shown by different papers presenting the findings of studies carried out in different Brazilian settings. Recently published studies tend to recruit larger samples of crack cocaine and/or snorted cocaine users rather than injection drug users (Additional file 1).

The majority of selected studies conducted multivariate analyses, and consistently identified injection drug use and syringe/needle sharing as key predictors of HIV-infection [53,59,60,64-66,69,71,72,74-76,81,83,85]. Key predictors of HIV-infection in the context of sexual risk behaviors included reporting to have had an HIV-positive or IDU sexual partner, engagement in sex work and male-to-male sex [39,53,59,64,65,67,69,72,81,83]. According to a few studies, a history of previous incarceration was found to be associated with HIV-infection [39,53,60,67]. Reports on infection by HCV and/or different sexually transmitted infections (STIs) have been identified by some authors as predictors of HIV-infection, but should rather be viewed as biomarkers of sexual and injection risk behaviors associated with both HIV and the acquisition of other STIs and blood-borne infections - additional file [53,67,81,83].

#### Meta-Analysis

Eight studies were excluded from our meta-analysis due to different reasons. The study conducted by De Boni and Pechansky [75] did not provide the HIV-prevalence for the overall sample and the study conducted by Guimarães et al. [78] with male inmates did not stratify their results according to different patterns of drug use. Two studies were conducted with participants who were co-infected with HIV and HCV, a specific subgroup that could bias the analysis toward people under higher risk and/or severely ill [65,66]. The study conducted by Caiaffa and colleagues [73] analyzed part of the dataset of AjUDE Brasil I and II projects, later published as comprehensive papers presenting the findings relative to the final dataset [67]. Four studies presented preliminary results on specific behaviors or genetic/virologic characteristics of drug users enrolled by the WHO Drug Injection Study Phase II, conducted in Rio de Janeiro [39,70,77,80]. Once again, we decided to include the most recent and complete analysis of this dataset [39]. In sum, while our systematic review analyzed 29 studies, we included only 22 studies in the meta-analysis.

Even after excluding studies defined as outliers, a large between-study heterogeneity remained (I^2 ^= 98.6%), with biased indicators and funnel plot asymmetry. In the sensitivity analysis, we were not able to ascribe the identified asymmetry to any specific study. Subgroup meta-analysis including only studies evaluating IDU remained highly heterogeneous (I^2^= 98.2%), as well as the pooled analysis of studies accessing only non-injection drug users (I^2 ^= 97.1%). When we stratified our studies according to incarcerated vs. non-incarcerated drug users, the heterogeneity remained (I^2 ^= 95.1% vs. 98.7%).

**Figure 6:**
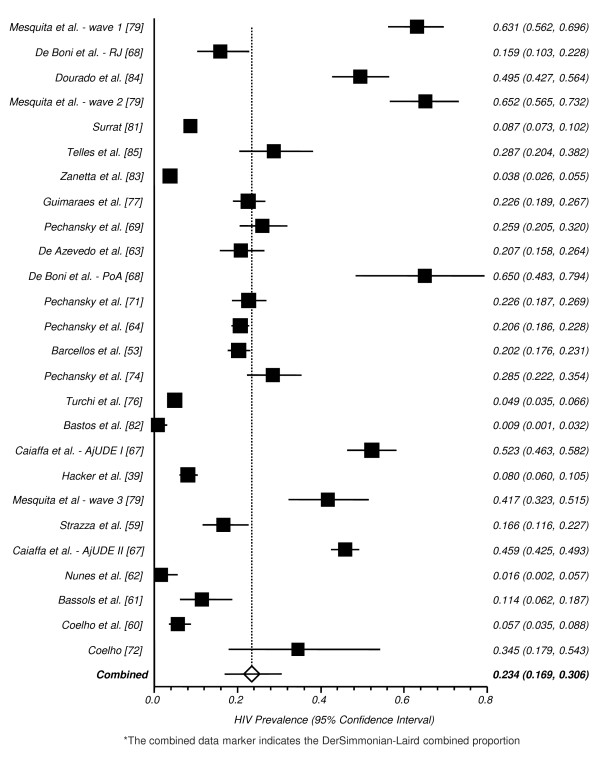
**Pooled Proportion of HIV Prevalence among DU/IDU**.*

The study conducted by Caiaffa et al [67] reports HIV-seroprevalence for two different multicenter studies (AjUDE Brasil I, conducted in 1998, and AjUDE Brasil II, conducted in 2000-01), therefore we divided such studies in our pooled analysis. The same strategy was used to pool the study from De Boni et al. [68] (one substudy was conducted in Rio de Janeiro and another in Porto Alegre), as well as the study published by Mesquita et al. [79] (presenting three different cross-sectional studies, conducted in 1991/92, 1994/96 and 1999). Studies were conducted in very different settings (drug addiction treatment centers, HIV-testing facilities and NGOs), accessed highly heterogeneous groups (e.g. female crack users vs. drug users under addiction treatment and street-recruited injection drug users) and were conducted over a long period of time (from 1991 to 2004), therefore compromising the reliability of our pooled analysis. Those caveats should be considered when analyzing the combined HIV prevalence across studies: 23.1 (95% CI: 16.7-30.2, Figure 6).

**Figure 7:**
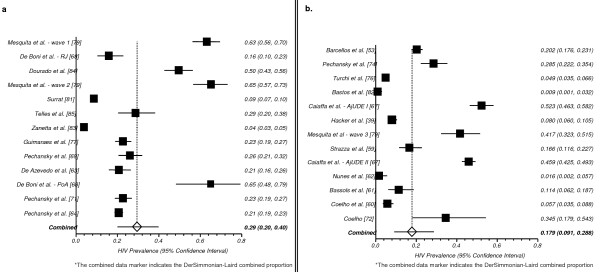
a. Pooled Proportion of HIV Prevalence among DU/IDU - Pre-HAART* b. Pooled Proportion of HIV Prevalence among DU/IDU - Pos-HAART*.

There has been discussion in the literature about whether people have changed their risk behaviors in the HAART era [1]. In an attempt to further contribute to this discussion, we aggregated studies according to period of data collection (pre-HAART era [until 1995] vs. pos-HAART era [since 1996]). Thirteen studies were conducted with IDU/NIDU in the pre-HAART era [63,64,68,69,71,77,79,81,83,85] and thirteen in the pos-HAART era [53,74,76,82,67,39,79,59,67,62,61,60,72]. Studies conducted in the pre-HAART period identified a higher HIV-prevalence: 29.5% (95% CI: 20.0 - 39.9) than studies conducted in the post-HAART period: 17.8% (95% CI: 9.09 - 28.8), see Figures 7a**and **7b, respectively.

A meta-regression model (Table 3) was fitted to evaluate major predictors of the between-studies heterogeneity, including the following covariates: "incarceration", drug using patterns (IDU vs. NIDU), "Brazilian region" (South vs. Southeast and Northeast), "study period" (1991-2001 vs. 2002-2004), and "recruitment site" (street recruited drug users vs. drug addiction or HIV testing facilities). After adjustment, only drug using patterns remained associated with the between-study heterogeneity (P-value < 0.0001). For studies accessing injection drug users, the AOR was 7.13 (95% CI: 2.52-20.20), when compared to studies evaluating non-injection drug users (Table 3).

**Table 3:** Covariates associated with "between-studies" heterogeneity according to multivariable logistic regression, Injection and non-injection drug users.

Variable	OR (95%CI)	AOR (95%CI)	
Incarceration			
Currently incarcerated	2.37 (0.58 - 9.70)	--------	
Non-incarcerated	1.00		
Drug use			
Injection drug use	5.57 (2.50 - 12.39)**	7.13 (2.52 - 20.20)**	
Non-injection drug use	1.00	1.00	
Brazilian Region			
South	1.86 (0.57 - 6.08)	--------	
Southeast and Northeast	1.00		
Study Period			
1991-2001	1.72 (0.32 - 9.30)	--------	
2002-2004	1.00		
Recruitment site			
Street recruited drug users	2.28 (0.83 - 6.25)*	0.68 (0.24 - 1.91)	
Drug addiction or VCT facilities	1.00	1.00	

Abbreviations: CI - Confidence Interval; OR - Odds Ratio;

AOR - Adjusted Odds Ratio (adjusted for all variables listed in the table)

* p-value = 0.10; **p-value < 0.0001

## Discussion

This is to our knowledge the first meta-analysis of HIV prevalence from three highly vulnerable populations carried out in Brazil: FSW, MSM and IDU/NIDU. Overall, the odds of having HIV infection are markedly and consistently higher among those populations than in the general population of adults of reproductive age. This latter has been stabilized around 0.6% since 2000 in Brazil [16,18,86].

There are a number of limitations to our study. FSW, MSM and drug users are often difficult to access and to enroll in surveys because of the social stigma associated with their behaviors and criminalization of drug use. These barriers may limited both the number and quality of studies identified, particularly those assessing FSW (n = 8) and MSM (n = 10). Drug users have been more systematically surveyed in Brazil, particularly due to surveys carried out in collaboration with international institutions, such as the World Health Organization (WHO) and the National Institute on Drug Abuse (NIDA/NIH). The majority of studies cited in this analysis used small convenience samples and were cross-sectional.

Gaps identified amongst the data and the resulting lack of accuracy of the pooled HIV/AIDS prevalence for MSM and FSW seem to be associated with the fragmentary character of sero- and behavioral surveillance targeting those populations in Brazil. This scenario is likely to be modified in the near future, with the implementation by the Brazilian Ministry of Health of biannual seroepidemiological studies targeting MSM, FSW and IDU/NIDU. The first round of such studies was conducted in 2009, but no peer-reviewed paper was published so far on such studies.

MSM, FSW and drug users tend to congregate in urban areas, at least partially explaining why the vast majority of reported studies are urban; again, this may limit the generalizability of our study findings, further complicated by the fact that most studies were carried out in large metropolitan areas of the most industrialized regions in Brazil. Publication bias tends to affect the results of meta-analyses, both in terms of clinical and public health research, and may compromise the accuracy of pooled measures [87].

To minimize the effect of publication bias, gray literature (e.g. national and international conferences and local reports) were included and researchers were directly contacted to obtain upcoming publications. We conducted a sensitivity analysis to assess each study impact on the pooled HIV-prevalence for each population group. Such an approach is important in assessing the validity of the assumptions made for the statistical calculations in meta-analyses [37,88]. Studies that may have an influence on the pooled analyses were removed. Results were then compared and a few studies were included only in the systematic review in order to control for potential biases. Unfortunately information on methodological quality of selected studies was insufficient to allow a detailed analysis of their quality.

Despite these limitations, this meta-analysis draws its strength from the pooled estimates for a large aggregate sample size of drug users (N = 13,063), while the aggregate sample size for FSW and MSM were not very large (N = 3,625 and 6,475, respectively). The small number of studies assessing FSW (N = 8) and MSM (N = 10), basically analyzing data from small-scale samples precluded further analysis and lead us to conclude that our meta-analysis for those two populations would not be conclusive [89].

Due to the significant heterogeneity identified in our pooled HIV prevalence for FSW (I^2 ^= 81.9), MSM (I^2 ^= 97.1%), and drug users (I^2 ^= 98.6%), our pooled HIV-prevalence is likely not to be valid as an accurate measure of risk. Subgroup analysis were not conducted for studies addressing FSW and MSM, due to the small number of studies addressing those populations and the lack of key variables that could guide such analyses. The subgroup analysis of studies on drug users highlighted high between-study heterogeneity. These trends of high HIV prevalence among FSW, MSM and IDU/NIDU speak in favor of the urgent need to improve targeted prevention strategies to those at-risk populations, particularly in the context of a developing country providing a comprehensive array of prevention interventions and HIV-treatment free of charge to any eligible individual living with AIDS.

The critical factor in making adequate HIV estimates in countries with low-level or concentrated epidemics is the availability of data. Countries that have serological and behavioral data for most at-risk groups may profit from better and more comprehensive estimates to inform policymaking and monitoring [90]. Exception made to the US, a few countries from Western Europe, and African countries where many different cooperative studies have been carried out assessing different populations and geographic areas such as Uganda, similar limitations as the ones identified by our group have been observed in most countries and have been associated with gross under and overestimates, such as the ones respecting India, later corrected by more recent studies [91].

Comprehensive information about vulnerable populations, behaviors that place people at risk, and knowledge of the current status and trends of infection rates among those populations are an essential component of sound programmatic decisions. In this sense, our pooled analyses may help to inform public policies but also to highlight the limitations of available data in Brazil.

The small number of studies on FSW and MSM, and the lack of key information that could help to better understand between-studies heterogeneity precluded meta-regression analyses for those subpopulations [38]. For studies addressing the drug using population, meta-regression analysis identified that injection drug users had a seven-fold increase in their risk to be HIV-infected, when compared to non-injection drug users. The measures of association among drug users seem to be consistent across injection and non-injection drug users, incarcerated and non-incarcerated participants, irrespectively of the geographic regions where they have been surveyed, the study period and the recruitment site. Those findings speak in favor of the external validity of many individual studies on drug using populations carried out in Brazil.

## Conclusions

The study results constitute a clear call to action on three fronts: surveillance, research, and prevention. The various analyses completed for this study may not necessarily explain complex differences in HIV epidemic dynamics, but bring additional evidence of high HIV prevalence rates among FSW, MSM, and drug users. HIV surveillance efforts should take into account the high burden of HIV among those vulnerable populations and expand surveillance strategies to access hidden populations. Social science, epidemiologic, and behavioral research should use standardized data collection tools to assess prevalence of HIV risk behaviors, knowledge about HIV, and social and sexual network interactions, therefore increasing our ability to cross-compare different studies. Ethnographic assessments could further explore the cultural and behavioral nuances of FSW, MSM, and drug users and help to refine data collection instruments. Advocacy and a non-discriminatory attitude may favor a better access to such hard-to-reach populations, fostering renewed HIV prevention, voluntary counseling and testing, and prompt referral to treatment.

Notably, there exists a risk that demonstrating high HIV prevalence rates among FSW, MSM and drug using populations will further contribute to the stigmatization already experienced by those populations. However, in Brazil (as in most countries), funds for prevention are generally allocated based on perceived needs; thus, the risk of increasing stigma must be balanced by the potential benefits of successfully advocating for dedicated funding resources for those populations and the agile referral to treatment in the context of universal access to HAART. Surveillance, research, and prevention efforts should work together to improve and disseminate currently available strategies targeting those populations in Brazil.

## Abbreviations

MSM: men who have sex with men; FSW: female sex workers; DU: drug users; PLWHA: people living with HIV/AIDS; IDU: injection drug users; NIDU: non-injection drug users; HAART: highly active antiretroviral therapy; BSS: behavioral surveillance surveys; CONSORT: Consolidated Standards of Reporting Trials; QUOROM: Quality of Reporting of Meta-analyses; MOOSE: Meta-analysis of Observational Studies in Epidemiology; TREND: Transparent Reporting of Evaluations with Nonrandomized Designs; CRISP: Computer Retrieval of Information on Scientific Projects; STIs: sexually transmitted infections

## Competing interests

The authors declare that they have no competing interests.

## Authors' contributions

MM and YL conducted literature search. MM, YL, FIB, MMFM evaluated the study articles and made decisions on inclusion and exclusion of the articles. MM, MMFM performed statistical analyses. All authors MM, MMFM, MBM, ARPP, YL, FIB were involved in the manuscript development and its revision. All authors read and approved the final manuscript.

## Pre-publication history

The pre-publication history for this paper can be accessed here:

http://www.biomedcentral.com/1471-2458/10/317/prepub

## Supplementary Material

Additional file 1 **"Table. Characteristics of selected studies and AIDS prevalence among drug users from Brazil, 1998-2009." **This file presents the characteristics of all selected studies describing AIDS prevalence among drug users from Brazil. Click here for file
