# Supplementary material for: HIV prevalence among female sex workers, drug users and men who have sex with men in Brazil: A Systematic Review and Meta-analysis
Source: BMC Public Health. 2010 Jun 7;10:317. doi: 10.1186/1471-2458-10-317 (PMC2898825; doi:10.1186/1471-2458-10-317)
Supplement: Additional file 1 — "Table. Characteristics of selected studies and AIDS prevalence among drug users from Brazil, 1998-2009." This file presents the characteristics of all selected studies describing AIDS prevalence among drug users from Brazil. [file 1471-2458-10-317-S1.DOC]

**Table.** Characteristics of selected studies and AIDS prevalence among drug users from Brazil, 1998-2009.

|  | |  |  |  | **Characteristics of Study Population** | | | **Drug abuse and HIV infection** | | |
| --- | --- | --- | --- | --- | --- | --- | --- | --- | --- | --- |
| **Source** | | **N** | **State** | **Design (Period)** | **Age [mean (range)]** | **Women (%)** | **Ethnicity (%)** | **Needle sharing** | **HIV prevalence** | Variables associated **with prevalent** HIV |
| Strazza et al. [59] | | 187[[1]](#footnote-2)  IDU: 21 | Sao Paulo | Cross-sectional (2000) | 31.0 ±9 (median: 29) | 100 | Caucasian: 156  Black: 51  Mulatto: 72  NA: 11 | 10 (43,5) | Overall: 16.6%  IDU: 38.1% | HIV-positive sexual partner:  OR: 6.9 (2.7 – 35.2);  IDU (previous 6 months):  OR: 3.3 (1.6 – 14.7);  Stable partner:  OR: 3.7 (1.5 – 8.3) |
| Coelho et al. [60] | | 333 male inmates  DU: 241  IDU: 29 | Sao Paulo | Cross-sectional (2003) | Mean; 30.1  Median: 28  Range: 19-69 | None | NA | Among IDU sample 37.9% | Overall:  5.7% (95%CI: 3.2-8.2) | Needle/syringe sharing  OR=7.63 (1.12-51.82)  Prison sentence >5 years  OR=4.48 (1.38-14.41) |
| Bassols et al.[61] | | 114  UD: 114 | RS | Case-control (2003-4) | 18-30 years: 52.1% | NA | NA | NA | Overall: 11.4%  DU: 11.4%  IDU: 12.5% | NA |
| Nunes et al. [62] | 125 crack users | | Salvador | Cross-sectional  (2001-02) | 22.5±6.2 | 100% | Afro descendents: 91.2% | NA | 1.6 (0.2-5.7) | NA |
| De Azevedo et al. [63] | | 241  109 IDU  132 DU | Campinas | Cross-sectional  (1995) | IDU: 27.5 ± 7.1  DU: 24.0 ± 6.1 | 2% | NA | 57.9% | IDU: 33.0  DU: 10.6 | NA |
| Pechansky et al. [64] | | 1449  IDU: 203  DU: 1246 | Porto Alegre, RS | Cross-sectional sequential  (1995-2004) | 29.0 ±10.1 | 539 (37.2) | NA | NA | Overall: 20.6%  IDU: 57.1%  Crack users:  26.5% | Injection drug use (previous 6 months):  AOR: 7.30 (5.10 – 10.40)  Homosexual men:  AOR: 3.04 (1.89 – 4.80)  Crack use:  AOR 2.03 (1.40-2.92). |

**Table 3.** Characteristics of selected studies and AIDS prevalence among drug users from Brazil, 1998-2009 (cont.)

|  |  |  |  | **Characteristics of Study Population** | | | **Drug abuse and HIV infection** | | | | |
| --- | --- | --- | --- | --- | --- | --- | --- | --- | --- | --- | --- |
| **Source** | **N** | **State** | **Design (Period)** | **Age [mean (range)]** | **Women (%)** | **Ethnicity (%)** | **Needle sharing** | **HIV prevalence** | | Variables associated **with prevalent** HIV | |
| Zocratto et al. [65] | 272 IDU | Brazil | Cross-sectional (1998) | 29.25 (±8.04) | 47 (17.3) | Caucasian: 49.5% | Previous 6 months  77,4% didn’t give used needle/syringes to others IDU  78,8% didn’t receive used needle/syringes | | 82.4% | | HIV+/HCV- MSM IDU:  AOR= 8.17 (2.67-25.02)  HIV+/HCV+ MSM IDU:  AOR=2.76 (1.32-5.78)  Syringe sharing: AOR=2.96 (1.50-5.86) |
| Silva & Barone [66] | 351 (HCV+)  89 IDU | São Paulo | Case control (1999-2001) | 18−29: 20.2%  30−39: 30.2%  40−49: 32.8%      >50: 16.8% | 43.3% | Non white: 23.6% | NA | | 74.2 | | Variables associated with HCV/HIV coinfection:  Any illicit drug use:  AOR=3.96 (1.55-10.13)  Needle or pipes sharing:  AOR=10.28 (4.00-26.42)  Women:  AOR=2.89 (1.16-7.08) |
| Caiaffa et al. [67] | 1144 IDU | Brazil | Cross-sectional  AjUDE Brazil I-1998  AjUDE Brazil II (2000-01) | AjUDE Brazil I: 29.2(±7.9)  AjUDE Brazil II:  28.5(±8.2) | AjUDE Brazil I: 17.5%  AjUDE Brazil II: 17.1% | Non white  AjUDE Brazil I 50.0%  AjUDE Brazil II  53.2% | Needle/syringe sharing (*lifetime*)  AjUDE Brazil I : 38.6%  AjUDE Brazil II 52.3% | | AjUDE Brazil I: 52.3%  AjUDE Brazil II: 45.8% | | AjUDE-Brazil I  IDU from high HIV prevalence sites (>50%)  AOR=2.16 (0.98-4.75)  HCV+: AOR=9.78 (9.73-40.19)  MSM IDU: AOR=2.09 (0.93-4.71)  AjUDE-Brazil II  IDU from medium HIV prevalence sites (10-50%):  AOR=10.66 (4.51-25.16)  IDU from high HIV prevalence sites (>50%):  AOR=31.69 (13.13-76.49)  Have been incarcerated:  AOR=1.40 (0.88-2.24)  HCV+ : AOR=15.47 (8.29-28.87)  IDU for ≥8 years:  AOR=2.12 (1.34-3.37)  MSM IDU: AOR=2.09 (1.27-3.16) |

**Table 3.** Characteristics of selected studies and AIDS prevalence among drug users from Brazil, 1998-2009 (cont.)

|  | |  |  |  | **Characteristics of Study Population** | | | **Drug abuse and HIV infection** | | |
| --- | --- | --- | --- | --- | --- | --- | --- | --- | --- | --- |
| **Source** | | **N** | **State** | **Design (Period)** | **Age** | **Women (%)** | **Ethnicity (%)** | **Needle sharing (%)** | **HIV Prevalence** | Variables associated **with prevalent** HIV |
| Hacker et al. [39] | 609 IDU | | Rio de Janeiro | Cross-sectional  (1999-2001) | “long-term” IDU  36.7 (mean)  “new injectors”:  27.5 (mean) | “long-term” IDU  9.1%  “new injectors”:  8.0% | Non white:  “long-term” IDU  56.3%  “new injectors”:  46.3% | “long-term” IDU  31.3%  “new injectors”:  44.2% | Overall: 8.0  “long-term” IDU: 11.7%  “new injectors”: 4.3% | *“New injectors”*  Homosexual practice  AOR= 8.03 (1.52 – 42.48)  *“Long-term injectors”*  Injection use with HIV+IDU  AOR= 3.91 (1.09-14.06)  Ever been in prision:  AOR= 2.56 (1.05-6.24) |
| De Boni et al. [68] | | 250  RJ: 146  PoA: 104 | Porto Alegre  e  Rio de Janeiro | Cross-sectional  Porto Alegre (95/97)  Rio de Janeiro (94/97) | RJ: 31 (mean)  Porto Alegre: 28 (mean) | RJ: 9,6%  Porto Alegre: 8,7% | NA | NA | Among 145 tested in RJ: 15,9%  Among 40 tested in PoA: 65% | NA |
| Pechansky et al. [69] | | 1,026  IDU: 106  DU:193 | Porto Alegre – RS | Cross-sectional (1995-1997) | Overall sample:  ≥ 25years: 60.6% | Overall sample:  43.6% | NA | NA | Overall sample:  15.1%  IDU: 50.0%  IDU & NIDU: 25.9% | Overall sample:  Male: AOR=1.8 (1.1-2.8)  ≥ 25years: AOR=1.7(1.1-2.7)  ≤3minimum wage: AOR=2.1(1.3-3.5)  IDU: AOR=7.6(4.4-13.0)  Sexual partner *might* be HIV+  AOR=1.8(1.1-3.2) |
| Teixeira et al. [70] | | 608 IDU | Rio de Janeiro | Cross-sectional  (1999-2001) | 35.3 (±8.1) | 18.7% | NA | NA | 7.89%  IDU: 9.2%  Ex-IDU:6.8% | NA |
| Pechansky et al.[71] | | 420 | Rio Grande do Sul | Cross-sectional  (NA) | <20: 16.3%  20-29: 45.0%  >29: 38.7% | 30.5% | NA | NA | 22.6% | >29 years:  AOR= 2.89 (1.17-7.12)  Up to 7 years of education:  AOR=2.10 (1.02-4.36)  Income <1 minimal wage  AOR=2.89 (1.32-6.32)  IDU since1980:  AOR=5.18 (2.89-9.28) |

**Table 3.** Characteristics of selected studies and AIDS prevalence among drug users from Brazil, 1998-2009 (cont.)

|  |  |  |  | **Characteristics of Study Population** | | | **Drug abuse and HIV infection** | | |
| --- | --- | --- | --- | --- | --- | --- | --- | --- | --- |
| **Source** | **N** | **State** | **Design (Period)** | **Age** | **Women (%)** | **Ethnicity (%)** | **Needle sharing (%)** | **HIV Prevalence** | Variables associated **with prevalent** HIV |
| Coelho [72] | 333 (Inmates)  IDU: 29  DU: 271 | Sao Paulo | Cross-sectional  (2003) | 30.06 (±8.18) | None | NA | 37.9% | Overall: 5,7%  IDU: 34.5% | MSM  AOR= 8.44 (0.73-22.21)  IDU  AOR=1.57 (0.31-3.23)  Needle sharing:  AOR=2.67 (0.55-5.00)  Sentence >5 years:  AOR=1.29 (0.10-2.48) |
| Caiaffa et al. [73] | 539  Porto Alegre (98): 137  Itajaí (98): 50  Porto Alegre (00/01): 255  Itajaí (00/01): 97 | Rio Grande do Sul  &  Santa Catarina | Cross-sectional  AjUDE Brazil I-1998  AjUDE Brazil II (2000-01) | Porto Alegre  1998: 28.5 ±8.4  2000/01: 31.4 ±8.6  Itajaí  1998: 32.5 ±7.4  2000/01: 27.4 ±8.6 | Porto Alegre:  1998: 15.3%  2000/01: 19.2%  Itajaí:  1998: 16.0%  2000/01: 12.4% | NA | At leasr once  Porto Alegre:  1998: 25.7%  2000/01: 58.5%  Itajaí  1998: 60.0%  2000/01: 36.8%  Previous 6 months:  Porto Alegre:  1998: 36.4%  2000/01: 39.1%  Itajaí  1998: 46.4%  2000/01: 52.2% | Porto Alegre:  1998: 48.5%  2000/01: 64.3%  Itajaí:  1998: 78.0%  2000/01: 30.9% | NA |
| Pechansky et al. [74] | 193 cocaine users | Rio Grande do Sul | Cohort (1996-98) | >25 years: 59.0% | 12% | Caucasian: 61.0% | NA | At baseline: 28.5% | ***At baseline***  <25 years old:  AOR=3.4(1.5-7.3)  >8 years formal education  AOR=2.6(1.1-5.8)  Single: AOR=3.9(1.7-8.9)  Irregular/no work  1.2 (0.6-2.6)  Cocaine injection (lifetime)  AOR=3.9(1.3-11.4) |

**Table 3.** Characteristics of selected studies and AIDS prevalence among drug users from Brazil, 1998-2009 (cont.)

|  |  | |  |  | **Characteristics of Study Population** | | | **Drug abuse and HIV infection** | | | |
| --- | --- | --- | --- | --- | --- | --- | --- | --- | --- | --- | --- |
| **Source** | **N** | | **State** | **Design (Period)** | **Age** | **Women (%)** | **Ethnicity (%)** | **Needle sharing (%)** | | **HIV Prevalence** | Variables associated **with prevalent** HIV |
| De Boni & Pechansky [75] | | 695 | Rio Grande do Sul | Cross-sectional  (NA) | 29.4 (±12.7) | 24.2% | NA | NA | | >20 years: 14.8%  20-30: 21.9%  <30 years: 27.0% | <30 years:  AOR=2.89 (1.17-7.12)  >7 years of education:  AOR=2.10 (1.02-4.36)  <1 minimum wage::  AOR=2.89 (1.32-6.32)  IDU since 1980:  AOR= 5.18(2.89-9.28)  Injection drug use (previous month):  AOR=4.30 (2.20- -8.83) |
| Turchi et al. [76] | | 839  (crack & snorted cocaine users) | Sao Paulo | Cross-sectional (97-98) | 26.9 (7.2) | 4.3 | NA | | NA | Overall: 4.9% (3.6-6.6)  IDU: 14.7 (9.6-21.6)  NIDU: 2.8 (1.8-4.4) | IDU: OR=6.0(3.0-12.0) |
| Barcellos et al. [53] | | 2,999  845 cocaine users | Rio Grande do Sul | Cross-sectional  (1996) | Overall sample:  < 20y: 15.9%  20-24: 24.0%  25-29: 17.9%  30-34: 15.2%  35-39: 10.8%  ≥ 40: 16.1% | Overall sample:  48.4% | Caucasian: 77.7% | | 53.0 | DU: 20.2%  IDU: 43.7% | Snorting cocaine:  AOR=2.43 (1.74-3.39)  Injecting cocaine:  AOR=4.45 (3.11-6.36)  Sharing needle equipment:  AOR=2.62 (1.38-4.98)  Inmate at juvenile center/prision:  AOR=1.53 (1.01-2.30)  Homosexual man  AOR=3.90 (2.16-7.05)  VDRL positive  AOR=3.46 (1.92-6.24)  Sex w/male prostitutes:  AOR=2.57 (1.57-4.01)  Sex w/HIV+ partner:  AOR=3.53 (2.24-5.56)  Sex w/IDU partner:  AOR=1.90 (1.32-2.73) |

**Table 3.** Characteristics of selected studies and AIDS prevalence among drug users from Brazil, 1998-2009 (cont.)

|  |  |  |  | **Characteristics of Study Population** | | | **Drug abuse and HIV infection** | | |
| --- | --- | --- | --- | --- | --- | --- | --- | --- | --- |
| **Source** | **N** | **State** | **Design (Period)** | **Age** | **Women (%)** | **Ethnicity (%)** | **Needle sharing (%)** | **HIV Prevalence** | Variables associated **with prevalent** HIV |
| Guimarães et al. [77] | 171[[2]](#footnote-3) | Rio de Janeiro | Cross-sectional  (1994-97) | 33.3±7.9 | 15.8% | NA | At least once (lifetime):  HIV/ HTLV negative:  60.8%  HIV or HTLV+  89.3%  HIV+: 88.4%  HTLV+: 92.3% | 26.9% | NA |
| Guimarães et al. [78] | 741 (Inmates)[[3]](#footnote-4)  Former-IDU: 145 | Sao Paulo | Cross-sectional  (1993-94) | 30.2 | None | White: 56.8%  Mulattos: 26.2%  Black: 16.9% | NA | 14.2% | NA |
| Mesquita et al. [79] | 457  1^st^ wave (N=214)  2^nd^ wave:  (N=135)  3^rd^ wave:  N=108) | Sao Paulo | Cross-sectional  1^st^ wave (1991/92)  2^nd^ wave: (1994/96)  3^rd^ wave (1999) | 25-40 years: 65% | 31% | NA | Previous 6 months:  1^st^ wave: 55%  2^nd^ wave:: 71%  3^rd^ wave: 24% | 1^st^ wave: 63.0%  2^nd^ wave:: 65.0%  3^rd^ wave: 42.0% | Women:  AOR=2.1 (1.3-5.0)  Syringe sharing:  AOR=2.7 (1.7-4.2)  More than 5 injections/day:  AOR=2.1 (1.3-3.3) |
| Albuquerque [80] | 246 | Rio de Janeiro | Cross-sectional  (1999-2000) | 29.59±0.59 | 7.7% | NA | Previous 6 months:  Never: 76,9%  Less than once a month: 15,4%  1-3 times a month: 6,5%  Around once a week: 1,2% | 6.1% | NA |

**Table 3.** Characteristics of selected studies and AIDS prevalence among drug users from Brazil, 1998-2009 (cont.)

|  |  |  |  | **Characteristics of Study Population** | | | **Drug abuse and HIV infection** | | | |
| --- | --- | --- | --- | --- | --- | --- | --- | --- | --- | --- |
| **Source** | **N** | **State** | **Design (Period)** | **Age** | **Women (%)** | **Ethnicity (%)** | **Needle sharing (%)** | **HIV Prevalence** | | Variables associated **with prevalent** HIV |
| Surrat [81] | 1,544 cocaine users  *Favela* sample: 855  Asphalt sample: 689 | Rio de Janeiro | Cohort  (1994-97) | Median  Overall: 29.0  *Favela*: 28.0  Asphalt: 30.0 | Overall: 22.5  *Favela*: 27.0  Asphalt: 16.8 | ***Overall***  Black: 34.1  White: 29.0  Multiracial: 36.8  ***Favela sample***  Black: 37.8  White: 22.6  Multiracial: 39.6  ***Asphalt sample***  Black: 29.6  White: 36.9  Multiracial: 33.2 | NA | At baseline: Overall: 8.7  *Favela*: 6.5  Asphalt: 11.3 | Asphalt residence:  AOR=1.530(1.06-2.22)  STD previous history  AOR=1.748(1.21-2.53)  Drug injection history  AOR=1.852(1.24-2.77)  Sex trading history  AOR=2.190(1.49-3.22) | |
| Bastos et al. [82] | 225 DU (treatment centers) | Rio de Janeiro | Cross-sectional (1998) | 32.1±9.5 | 13.3% | NA | NA | 0.9 | NA | |
| Zanetta et al. [83] | 1199 juvenile inmates[[4]](#footnote-5)  Cocaine users: 755 | Sao Paulo | Cross-sectional (1994-95) | 16.2 [12-21] | 7.6% | NA | ♂: 38.0%  ♀: 73.0% | Overall: 2.9%  ♂: 2.6%  ♀: 10.3%  Cocaine users: 3.8% | Female  >5sexual partners in life:  OR=2.77 (0.54-14.10)  Commercial sex work:  OR=5.98 (1.04-34.30)  Currently reported STD:  OR=4.06 (0.79-20.90)  Male  HCV infection:  OR=26.5 (8.83-79.70)  Age ≥ 18 y:  OR=3.45 (1.21-9.86))  Injection drug use:  OR=3.39 (1.10-10.40) | |

**Table 3.** Characteristics of selected studies and AIDS prevalence among drug users from Brazil, 1998-2009 (cont.)

|  |  |  |  | **Characteristics of Study Population** | | | **Drug abuse and HIV infection** | | |
| --- | --- | --- | --- | --- | --- | --- | --- | --- | --- |
| **Source** | **N** | **State** | **Design (Period)** | **Age** | **Women (%)** | **Ethnicity (%)** | **Needle sharing (%)** | **HIV Prevalence** | Variables associated **with prevalent** HIV |
| Dourado et al. [84] | 216 | Bahia | Cross-sectional  (1994-96) | ♂: 24.6 (mean)  ♀: 27.4 (mean) | 18% | NA | NA | Overall: 49.5%  ♂: 44.1%  ♀: 74.4% | NA |
| Telles et al. [85] | 108[[5]](#footnote-6) | Rio de Janeiro | Cross-sectional  (1994-96) | 33.7±7.9 | 15.5% | NA | Previous 6 months:  56.5%  Previous 5 years:  73.8% | 28.7% | Live in low income areas:  AOR=5,57 (1,39 - 22,27)  No regular income source:  AOR=3,26 (1,01 - 10,51)  1^st^ injection drug use with < 18 years  AOR=2,50 (0,99 - 6,28)  Recruited on “streets”  AOR=7,91 (1,39 - 22,27)  Needle/syringe sharing (previous 6 months):  AOR=4,41 (1,33 - 14,64) |

1. Female inmates who are drug users [↑](#footnote-ref-2)
2. Participants tested for HIV-infection [↑](#footnote-ref-3)
3. Participants tested for HIV-infection with conclusive results [↑](#footnote-ref-4)
4. Participants tested for HIV-infection [↑](#footnote-ref-5)
5. Participants tested for HIV-infection [↑](#footnote-ref-6)
